# Transactions of the Michigan State Medical Society

**Published:** 1889-02

**Authors:** 


TRANACTIONS OF THE MICHIGAN STATE MEDICAL SOCIETY. Twenty-third Annual meeting, held in Detroit, June, 1888. This volume contains 364 pages, and was printed by Gulley, Bowman & Co,. Detroit.

The retiring president, Theadore A. McGraw, M. D., of Detroit, in his address on "The Dependence of the Profession on the People" makes a very sensible remark, when he says that the people must be educated, and that "the habit of discussing professional matters of public interest in the daily prints must be recognized as one that is proper and right for professional men." Simeon S. French, M. D., of Battle Creek, is President, and Geo. Duffield, M. D., of Detroit, Secretary.—B.

